# Native T1 myocardial tissue characterisation in patients with pulmonary hypertension: findings from International T1 Multicentre Study

**DOI:** 10.1186/1532-429X-17-S1-O32

**Published:** 2015-02-03

**Authors:** Andrew J Swift, David D'Cruz, Shirish R Sangle, John G Coghlan, Andrew Jabbour, Chung-Yao Yu, Anne Keogh, David M Higgins, Eike Nagel, Valentina Puntmann

**Affiliations:** 1 Department of cardiovascular Science, University of Sheffield, Sheffield, UK; 2 Cardiovascular Imaging Department, King's College London, London, UK; 3 Guys and St Thomas' NHS Trust, London, UK; 4 University College London, London, UK; 5 St Vincent's Hospital and The Victor Chang Cardiac Research Institute, Syndey, UK; 6 Philips Healthcare, Guilford, UK

## Background

Pulmonary hypertension is a severe disorder characterized by elevated pulmonary artery pressure leading to right ventricular (RV) failure and premature death. We have recently shown that high signal in septal myocardium by cardiovascular magnetic resonance (CMR) late gadolinium enhancement (LGE) imaging indicates more severe disease. This study investigated whether markers of interstitial septal changes by T1 mapping relate to parameters of biventricular function and structure and makers of disease severity in pulmonary hypertension.

## Methods

Patients with a suspected or established diagnosis of pulmonary hypertension, based on a previous echocardiography or catheterisation, respectively, and control subjects underwent routine clinical CMR protocol (1.5 and 3 Tesla) and T1 mapping prior to and >20 minutes after administration of 0.2 mmol/kg of gadobutrol. T1 values were measured in mid-ventricular slices conservatively within septal myocardium. To transform native T1 values into a binary variable (normal/abnormal), the established cut-offs of >990ms at 1.5T or >1090ms at 3T, respectively, were used. For comparison of two and more than two normally distributed variables, Student's t-test and one-way analysis of variance (ANOVA, with Bonferroni's post-hoc test) as appropriate. Associations were explored by single and multivariate linear regressions.

## Results

**Figure 1:**
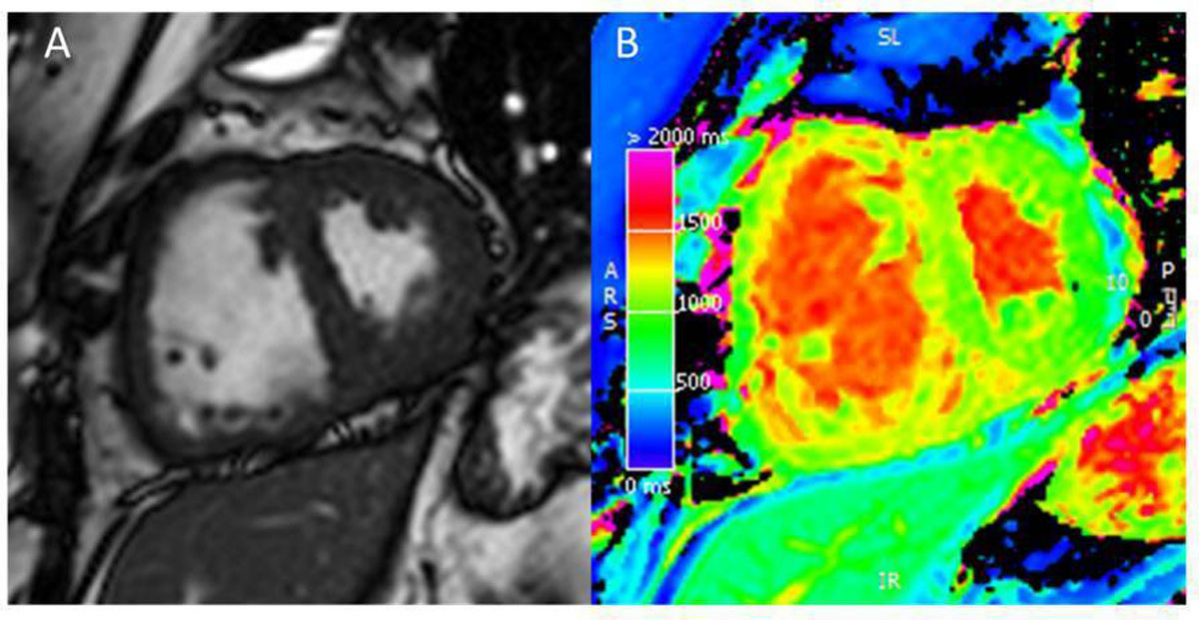
Shows a systolic short axis mid chamber bSSFP image (A), and native T1 parametric map (B) in a patient with pulmonary hypertension.

17 patients with pulmonary hypertension, 13 patients with suspected pulmonary hypertension and 40 healthy controls (20 at 1.5T and 20 at 3T) were identified. Of the patients with pulmonary hypertension, seven had pulmonary arterial hypertension (PAH), two had pulmonary hypertension owing to left heart disease, three had pulmonary hypertension associated with lung disease and/or hypoxemia and five patients had chronic thromboembolic pulmonary hypertension. Figure 1 illustrates native T1 values in a patient with PAH with septal flattening and visible elevated T1 at the RV insertion point. Compared to controls, both patients groups had higher T1 values (suspected PH (p=0.002), established PH (p=0.023)), at 1.5T strengths. At 3T only patients with established pulmonary hypertension have higher T1 values than controls, p=0.017. A greater proportion of patients with established pulmonary hypertension had an abnormal native T1 compared to patients with suspected pulmonary hypertension. Elevated native T1 values were associated with reduced LV end diastolic volume (p=0.009), reduced LV stroke volume (p=0.017) and interventricular septal (IVS) angle (p=0.004), but were not significantly associated with RV remodelling. At multivariate analysis high IVS angle was independently linked to elevated septal T1 values.

## Conclusions

The findings reveal the presence of interstitial myocardial changes by native T1 in patients with pulmonary hypertension, which are closely associated with LV remodelling and ventricular interaction. Native T1 may become a potential novel marker for the assessment of disease severity in pulmonary hypertension.

## Funding

Department of Health via the National Institute for Health Research (NIHR) comprehensive Biomedical Research Centre award to Guy's & St Thomas' NHS Foundation Trust in partnership with King's College London and King's College Hospital National Health Service Foundation Trust. Supported by the King's BHF Centre of Research Excellence.

